# Experimental Study of Radial Distortion Compensation for Camera Submerged Underwater Using Open SaltWaterDistortion Data Set

**DOI:** 10.3390/jimaging8100289

**Published:** 2022-10-19

**Authors:** Daria Senshina, Dmitry Polevoy, Egor Ershov, Irina Kunina

**Affiliations:** 1 Evocargo LLC, 129085 Moscow, Russia; 2 Moscow Institute of Physics and Technology (National Research University), 141701 Dolgoprudny, Russia; 3 Smart Engines Service LLC, 117312 Moscow, Russia; 4 Federal Research Center “Computer Science and Control” of Russian Academy of Sciences, 119333 Moscow, Russia; 5 National University of Science and Technology MISIS, 119049 Moscow, Russia; 6 Institute for Information Transmission Problems of Russian Academy of Sciences, 127051 Moscow, Russia

**Keywords:** underwater shooting, radial distortion, camera calibration, refractive index

## Abstract

This paper describes a new open data set, consisting of images of a chessboard collected underwater with different refractive indices, which allows for investigation of the quality of different radial distortion correction methods. The refractive index is regulated by the degree of salinity of the water. The collected data set consists of 662 images, and the chessboard cell corners are manually marked for each image (for a total of 35,748 nodes). Two different mobile phone cameras were used for the shooting: telephoto and wide-angle. With the help of the collected data set, the practical applicability of the formula for correction of the radial distortion that occurs when the camera is submerged underwater was investigated. Our experiments show that the radial distortion correction formula makes it possible to correct images with high precision, comparable to the precision of classical calibration algorithms. We also show that this correction method is resistant to small inaccuracies in the indication of the refractive index of water. The data set, as well as the accompanying code, are publicly available.

## 1. Introduction

Various kinds of aberrations occur in optic systems. Therefore, when shooting with a camera other than a pinhole one, distortion in the image may emerge, such as radial distortion (RD) [1]. RD can be eliminated by calibration of the camera, during which the parameters are estimated for further software distortion compensation.

In the air, the refractive index (and, hence, the distortion parameters) barely changes, which allows the camera calibration to be carried out only once to obtain undistorted images. However, after submersion of the camera underwater, the distortion parameters change significantly due to the refraction of light waves when passing through the water–glass–air interfaces.

The exploration of RD correction methods underwater and study of their characteristics are relevant among scientists in many applied scientific fields. Underwater video analytics systems, for instance, are being developed to monitor the population status of forage fish in closed reservoirs [2]. The quality of the RD correction turns out to be crucial for the high-quality performance of such a system. A related problem has been described in the work [3], where a computer vision system was suggested as an alternative to the use of electric fishing rods for spawning fish “sampling”. The authors noted that an important factor affecting the quality of its operation is proper underwater RD correction. The author of [4] came to similar conclusions, in the context of an underwater object tracking task. Furthermore, in the work [5], the authors noted the importance of RD correction for the study of fish behaviour in their natural environment.

The authors of the work [6] focused on a system for autonomous mapping of the ocean floor, and demonstrated a number of experimental results using real and synthetic data. Based on these results, they explicitly showed the strong affect of even small errors in the estimated RD parameters on the constructed map quality. The same problem has been faced by scientists during the creation of a system for three-dimensional reconstruction of cultural heritage objects underwater [7]. The task of eliminating RD caused by the submersion of a lens underwater has also arisen in a work devoted to the creation of an visual odometry algorithm for an underwater autonomous vehicle [8].

A study that particularly deserves mentioning is [9], in which the authors encountered an underwater RD calibration problem in the task of improving the image aesthetics. Often, to enhance the image, scene depth map restoration is performed; for example, it is necessary to simulate the bokeh effect. The resulting distorted structure of the depth map can, to the contrary, lead to a decrease in aesthetics. Moreover, manufacturers of mobile phones and smartphones are currently extensively switching to the new IP68 [10] standard, with increased requirements for the stability of underwater performance, which has leads to growth in the popularity of amateur underwater photography.

The classic approach [11] for underwater RD correction is repeating calibration after submersion, which is a rather expensive and time-consuming process: a set of images of a special calibration object from different angles must be collected, followed by performing an automated search for RD correction parameters, the quality of which is determined by the prepared set of calibration images.

**Figure 1:**
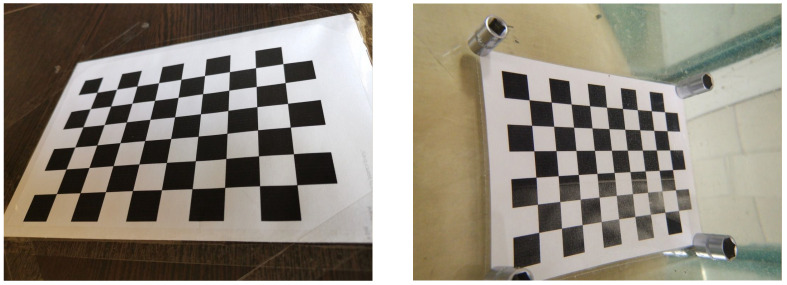
Examples of images made with the same camera in the air (**left**) and underwater (**right**).

Most modern mobile phones cameras are sufficiently well pre-calibrated for shooting in the air, where RD is typically almost absent. In contrast, when underwater distortion appears, recalibration is required (Figure 1); however, due to the high labour consumption and complexity, its execution is not always justified.

Although it has been practically shown that such a shooting protocol actually allows for underwater RD elimination with sufficient quality [12], it is not applicable to any shooting scenario. For example, during amateur photography, strict limitations on the underwater shooting session time, or cases when operator’s expertise is limited, such an approach will generally not work. The average mobile phone user is more likely to delete a distorted image than figure out how to fix it.

The problem of RD correction parameter estimation is also aggravated by the fact that the refractive index may differ in different water reservoirs; consequently, the correction parameters found for some reservoir may not be suitable for another. The same may be true for a single reservoir, if its salinity or the temperature of the water changes [13]. It turns out that either the camera must be recalibrated for every submersion, or the necessity of recalibration must somehow be assessed.

Thus, the question arises: “Is it possible not to recalibrate, but to recalculate the parameters of the RD correction, knowing the refractive index of water?” For the first time, the answer to this question was proposed in [14]. In this study, the authors conducted a number of experiments, on the basis of which they derived an empirical formula for radial distortion correction depending on the refractive index. Later, in the work [15], the authors presented similar results, which were obtained theoretically, not experimentally.

In addition to deriving the RD correction formula, Ref. [15] have demonstrated its accuracy for a real image. However, there remain a number of questions, the answer to which requires a full-fledged experiment:

- How accurate is such analytical calibration?
- How does the accuracy of the initial calibration in the air affect the RD correction quality for this type of water?
- How does the inaccuracy of water refractive index selection affect the RD correction quality?

This work is devoted to answering these questions, thereby assessing the practical applicability of the RD compensation formula proposed in [15].

However, to answer these questions, a data set of underwater images which allows us to numerically assess the quality of distortion correction is required; for example, a set of images of some calibration object with a known structure. We could not find such an open data set. Therefore, a new set of images of a chessboard with manually marked cells square was assembled, called SWD (Salt Water Distortion), which will be described in detail in Section 4 of this work.

The new results of numerical experiments confirming the practical applicability of the RD correction formula are described in Section 5.1 and Section 5.2. Section 5.3 evaluates the quality of the performance of an automatic detector of chessboard cell corners in underwater images, used as a tool to assess the quality of associated algorithms.

## 2. Reference Calibration Algorithm

In the work [16], it has been shown that the use of the pinhole camera model underwater has some restrictions, as the refractive index underwater depends on the wavelength. However, according to [17], for 19 °C distilled water, the refractive index varies from 1.332 at a wavelength of 656 nm to 1.343 at a wavelength of 404 nm. As such variation is insignificant, the resulting angular difference in refraction is insignificant. The authors of [2] came to a similar conclusion.

An alternative to using the pinhole camera model is a more complex model, the so-called non-single viewpoint model (nSVP), while all falling rays pass through one point in the traditional camera model, in nSVP, they fall on the caustic curve [18]. As the authors of [19] have mentioned, the standard calibration object (chessboard) is not suitable in this case. Instead, a new calibration object was proposed: a perforated lattice, which is illuminated by two different wavelengths of light, forming an exactly known scheme of point light sources.

Thus, as the pinhole camera model is quite accurate, and the complications of the model lead to significant complication of the calibration procedure and the data collection process, only RD correction algorithms based on the classical pinhole camera model were studied in this work.

In this category, there exist many algorithms for RD calibration [20], which can be divided into the following groups:

- Calibration algorithms calculating RD correction parameters based on a series of images of a calibration object; for example, a chessboard [11], a flat object with evenly spaced LED bulbs [21], or an arbitrary flat textured object [22].
- Calibration algorithms based on active vision, calculating the parameters of RD correction from a series of scene images; in this case, information about camera movement is known during calibration [23].
- Self-calibration algorithms that calculate the RD correction parameters by checking the correctness of the epipolar constraint for a series of images of the same scene taken from different angles [24].
- Self-calibration algorithms that calculate camera parameters from a single-shot image [25,26].

As the purpose of this work is to check the formula for RD parameter re-calculation in laboratory conditions, the algorithm described in [11] is considered in our work as the most suitable reference; hereafter, we denote this algorithm by “classic”.

It is worth mentioning that, while the chosen classic algorithm aims to address more complex problems (i.e., not only RD correction), this algorithm is used only as a reference and to have an adequate baseline for quality assessment of the RD correction formula.

## 3. Methods

### 3.1. Classic Algorithm for Radial Distortion Correction

The classic algorithm works with a set of images of a flat calibration object with periodic structure (e.g., a chessboard) and known geometric parameters, taken from various angles. In these images, special points are defined (e.g., the nodes of a chessboard). This method is a traditional approach, in which the calculation of parameters of the distortion model is performed simultaneously as the calculation of the internal parameters of the camera (in the pinhole camera model), through solving the a non-linear optimization problem.

The calibration algorithm typically uses a standard distortion model [27] describing radially symmetric distortions. The software implementation of this algorithm in the OpenCV [28] library uses a model in which the transformation of the image point coordinates (in the plane of the pinhole camera screen) is set as follows:

(1) $$\left[\begin{array}{c} x^{\prime }\\ y^{\prime } \end{array}\right]=\left[\begin{array}{c} x\\ y \end{array}\right]\frac{1+k_{1}r^{2}+k_{2}r^{4}+k_{3}r^{6}}{1+k_{4}r^{2}+k_{5}r^{4}+k_{6}r^{6}},$$

where ${\left(x\;y\right)}^{T}$ denotes the initial image point coordinates; ${\left(x^{\prime }\;y^{\prime }\right)}^{T}$ denotes the coordinates of this image point after distortion correction; $r=\sqrt{x^{2}+y^{2}}$; and $k_{1},\ldots ,k_{6}$ are the distortion coefficients.

### 3.2. Formula for Radial Distortion Correction

In [15], it was shown that, with a known refractive index of water, the correction of the distortion occurring when the camera is immersed can be described by converting the coordinates of the image in the plane of the pinhole camera screen, as follows:

(2) $$\left[\begin{array}{c} x^{\prime \prime }\\ y^{\prime \prime } \end{array}\right]=\frac{1}{\sqrt{1-\left(n^{2}-1\right)r^{2}}}\left[\begin{array}{c} x^{\prime }\\ y^{\prime } \end{array}\right],$$

where ${\left(x^{\prime }\;y^{\prime }\right)}^{T}$ and ${\left(x^{\prime \prime }\;y^{\prime \prime }\right)}^{T}$ denote the coordinates of the object on the pinhole camera screen when shooting in the air and underwater, respectively; $r=\sqrt{{\left(x^{\prime }\right)}^{2}+{\left(y^{\prime }\right)}^{2}}$; and *n* is the refractive index of water.

This formula is obtained by assuming infinitesimal thickness of the material separating water and air.

## 4. Calibration Data Set of Images in Salt Water (SWD)

Shooting was conducted using a modern mobile phone (Huawei Mate 20 Pro). The data set was obtained using two cameras: a telephoto (focal length 7.485 mm) camera and a wide-angle (focal length 2.35 mm) camera. The image size was the same for both cameras, and was equal to 1459×1094 pixels. A 13×9.1 centimetre chessboard was used as a calibration object, with a side length per chessboard cell of 1.3 cm (10×7 cells).

Underwater image collection was carried out by submerging the cameras into an aquarium filled with tap water (salinity less than 1%). The non-salty water image set contained 48 and 56 images for each camera, correspondingly. Then, the salinity was increased, using table salt, to 13, 27, or 40%, with the required amount of salt having been pre-calculated, as the spatial measurements of the used aquarium were known. The obtained salt water image sets consisted of 34, 86, and 88 images for the telephoto camera and 47, 89, and 80 images for the wide-angle camera, respectively.

**Figure 2:**
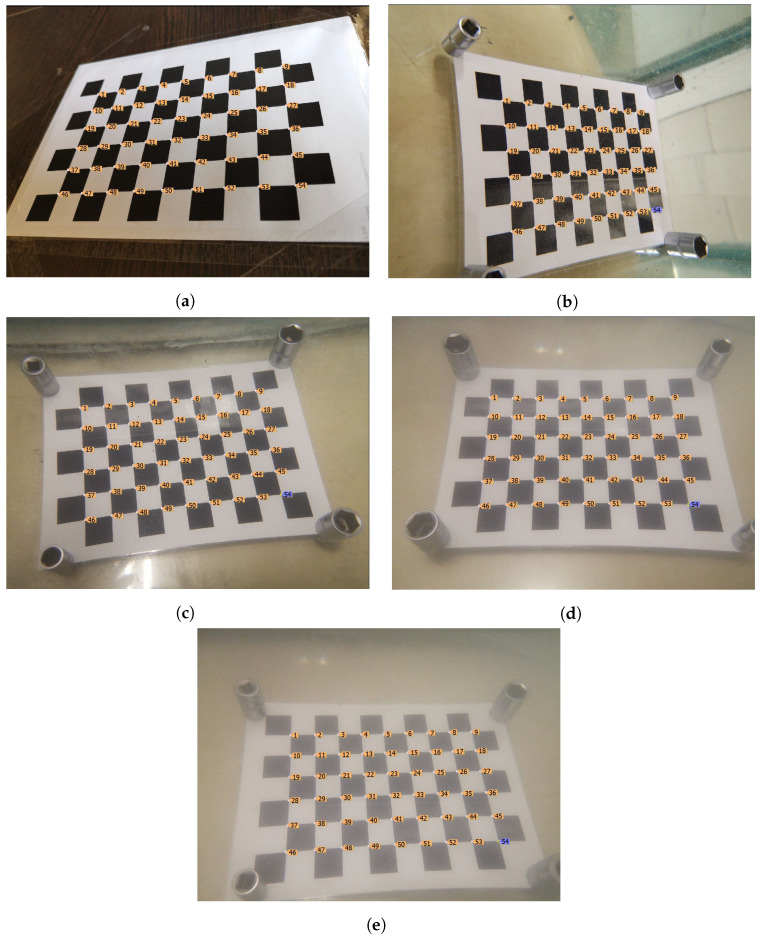
Example images from SWD data set and cell corner markup: (**a**) Captured in the air; (**b**) captured underwater (salinity <1%); (**c**) captured underwater (salinity 13%); (**d**) captured underwater (salinity 27%); and (**e**) captured underwater (salinity 40%).

The coordinates of the cell corners of the chessboard image for all collected images were marked manually. The coordinates of each point were specified with sub-pixel accuracy and, depending on the situation, either a point in the middle of a pixel, a point on a pixel grid, or a middle point of the border of two adjacent pixels was assigned to the corner pre-image of the chessboard. Examples of images and the used markup are provided in Figure 2. The collected data set is publicly available (https://github.com/Visillect/SaltWaterDistortion (accessed on 11 October 2022)).

**Figure 3:**
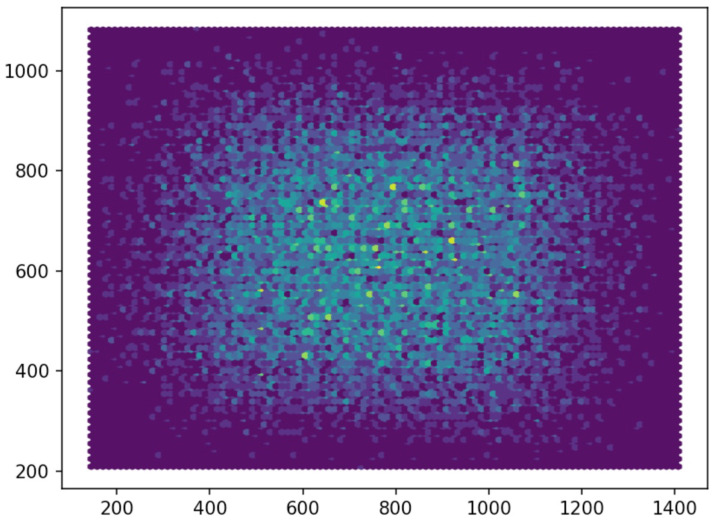
Histogram of the corner distribution over the image area in the SWD data set for the telephoto camera.

It is also worth noting the technical difficulties that arose during the collection of the data set, which affected its appearance:

- After the shutter of the smartphone camera was released, the image was processed programmatically from the RAW format, as a result of which the final image was cropped, which was not displayed on the smartphone screen at the time of shooting. Thus, it was difficult to obtain a board at the edge of the image, where the distortion is known to be maximal. An example histogram of the distribution of board cells corners in the original image is shown in Figure 3.
- Due to the movement of water movement during shutter release and further image processing, many photos turned cloudy. These photos had to be excluded as, due to the turbidity, some cell corners of the chessboard became indistinguishable.
- With an increase in salinity, the water turbidity also increased, as the salt used was not pure enough and contained impurities.
- Laminated paper with the chessboard image was used for shooting underwater. Because of this, light glares appeared at some angles. Particularly, it became difficult to determine the location of the chess grid corners, and such photos were also manually excluded.

## 5. Experimental Results

### 5.1. Precision of the Correction Formula

In this work, all numerical experiments were carried out on images captured only by a telephoto smartphone camera.

The initial calibration of the smartphone camera was carried out in air, using the classical method with images taken in the air from the SWD data set. Thus, the original RD of the lenses was eliminated, and the associated correction parameters were fixed. For images corrected with these parameters, the RD correction formula with varying index *n* was applied to the underwater photography. The marked points on the chessboard for all images in the experiment were also re-calculated, according to these parameters, using Formula (1). Based on analysis of the structure of the transformed set of marked points, the precision of the correction was evaluated.

To correctly compare the results of such correction with the classical method, the RD correction parameters underwater were calculated using the classical method for each degree of salinity separately. After that, using the obtained RD correction parameters, the marked points were transformed. Similarly to above, the precision of the correction algorithm was evaluated based on the obtained results.

**Figure 4:**
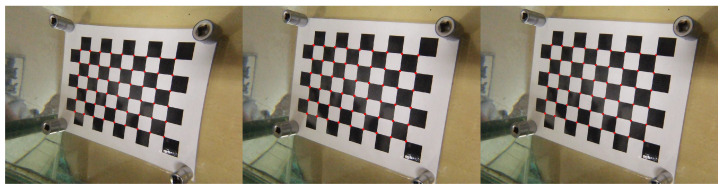
Examples of images (from left to right): Taken underwater (salinity <1%) with parameters for correcting radial distortion when shooting in air; corrected using the classical calibration method; and corrected by Formula (2).

In this paper, a software implementation presented in the OpenCV [28,29] library was used for the classical calibration method. An example of image correction using both methods is shown in Figure 4.

The experiment was carried out the same way for each level of salinity. Optimal distortion correction coefficients were selected through cross-validation (the size of the training set was 75%, and the validation set was 25%) on the training set (i.e., with the exception of 10 test images).

To assess the quality of the radial distortion effect correction, the structure of the transformed set of marked points was analysed: the better that the points corresponding to one straight line of chessboard corners fit to a straight line, the better the calibration effect. In this paper, two metrics were used to estimate the quality of calibration:

- Metric 1. The standard deviation of the cell corners from the straight line approximating them, determined using the OLS method, was estimated.
- Metric 2. The distance from the straight line constructed through the corners of the chessboard and the most distant cell corner corresponding to it.

The second metric is especially important for quantifying the effect, as it is more likely that the furthest line will be at the border of the image, where RD correction errors are particularly pronounced.

For each set of images corresponding to different salinity indices, four errors were calculated:

- M1. The average value of metric 1 on the entire set of images for all lines.
- M2. The maximum value of metric 1 on the entire set of images among all lines.
- M3. The average value of metric 2 on the entire set of images for all lines.
- M4. The average value of metric 2 on the entire set of images for the most distant lines (for each image, one such line was chosen).

The point coordinates were multiplied by the same number, such that the length of the largest chessboard side for each image was 1000 pixels. This normalization was carried out to eliminate the influence of the scaling factor when correcting the image. The results of the experiment are presented in Table 1.

It can be concluded, from the table, that the errors in the air after applying the classical method differed only by a few hundredths of a pixel from the errors before applying the method, which is reasonable as most of cameras (and especially smartphone cameras) are designed and optimised for shooting in the air. At the same time, errors in water at all levels of salinity, after applying the classical calibration method and the correction Formula (2), were much lower than the errors obtained before RD correction. The correction precision of both methods was comparable; in some cases, the correction error using Formula (2) turned out to be even lower than that obtained when using classical method. From this, we can conclude that the proposed formula is applicable for the correction of RD that occurs when a camera is submerged underwater.

### 5.2. Dependence of the Correction Precision on Salinity

As is well-known, the refractive index of water is affected by salinity and temperature [13]. In this paper, to conduct experiments with different refractive indices, the degree of salinity of water at room temperature was varied. The salinity of water is easier to control technically; moreover, it influences the refractive index of water more significantly.

Four sets of underwater images with a telephoto camera from the SWD data set collected in water with different salinity levels were used for the experiment. The refractive index n=1.33 corresponds to distilled water, while n=1.40 corresponds to 40% saline solution (i.e., the salinity of the Dead Sea) [30]. The 13% and 27% salt solutions corresponded to n=1.35 and n=1.37, respectively.

For each of these sets, correction was performed using Formula (2) with different values of the specified refractive index. The results of the experiments are presented in Table 2. The experiment showed that even significant changes in the salinity index only slightly affected the precision of the final correction; namely, the precision did not change by more than 0.2.

It should also be noted that, for all experiments, the error increased with an increase in the refractive index. This was due to the imperfection of the image normalization method: with an increase in the refraction parameter, the degree of image distortion increases, which leads to an increase in the final error when normalizing the largest chessboard side to a size of a thousand pixels.

From the results of this experiment, it can be concluded that the correction of radial distortion by Formula (2) with a refractive index of 1.33 provides acceptable precision, in most cases.

### 5.3. Quality of the Automatic Chessboard Cell Corner Detector

To automatically assess the quality of the RD correction algorithms, it was necessary to be able to programmatically search for cell corners in the calibration object (i.e., the chessboard). This feature was provided by the findChessboardCorners function in the OpenCV library. A logical question arises: “Is it possible to use this function to assess the quality of correction in experiments with underwater images without using a data set?”.

To answer this question, it was considered sufficient to compare the detection result with the marked points in the SWD data set. For each image, the largest Euclidean distance between a pair of corresponding points was estimated, the comparison results are presented in Table 3.

The results clearly demonstrate that the accuracy of the cell corner detector was significantly lower than the accuracy of correction. Large values in the column with maximum errors indicate that there were outliers that made at least the M2 and M4 metrics uninformative. Finally, the results in the table indicate that, with increasing salinity and turbidity of the water, the reliability of this measurement method decreases.

## 6. Conclusions

In this article, we described a new open data set for evaluating the accuracy of underwater radial distortion calibration algorithms under different refractive indices. The data set consists of 662 images of a chessboard collected with two different cameras, with the location of cell corners marked manually.

Based on the collected data set, a number of experiments were conducted to assess the practical applicability of a radial distortion correction formula when the camera is submerged underwater. According to the experimental results, the precision of RD correction using the formula was not inferior to a full-fledged calibration procedure for specific operating conditions. We also showed that the inaccuracy of specifying the refractive index of water does not significantly affect the precision of the correction and, so, it can be set equal to 1.33.

Thus, this article experimentally confirmed that the use of the radial distortion correction formula allows us to not only significantly simplify and reduce the cost of operating a camera underwater, but also maintains the calibration accuracy at a sufficient level.

## Figures and Tables

**Table 1:** Comparison of the precision of radial distortion correction using the classical method and Formula (2).

Conditions	Method	M1	M2	M3	M4
In the air	Without correction	0.51	2.74	1.06	1.66
	Classic	0.49	2.67	1.02	1.59
Underwater (salinity <1%)	Without correction	1.41	7.57	2.45	3.97
	Classic	0.93	4.89	2.33	3.68
	Formula (2) (*n* = 1.33)	0.65	3.96	1.79	2.75
Underwater (salinity 13%)	Without correction	1.51	6.80	2.79	5.28
	Classic	0.90	4.38	2.13	3.49
	Formula (2) (*n* = 1.35)	0.69	4.05	1.80	2.64
Underwater (salinity 27%)	Without correction	1.60	8.54	3.25	5.36
	Classic	1.03	4.79	2.66	3.90
	Formula (2) (*n* = 1.38)	0.91	4.55	2.44	3.54
Underwater (salinity 40%)	Without correction	1.50	7.03	3.54	6.08
	Classic	0.89	5.24	2.34	3.63
	Formula (2) (*n* = 1.40)	0.83	5.00	2.18	3.24

**Table 2:** Dependence of RD correction error when using Formula (2) on the accuracy of specifying the water refraction index.

The Set of Images	Assumed n	M1	M2	M3	M4
Underwater, salinity <1% (actual refractive index n=1.33)	1.33	0.6504	3.9616	1.7934	2.7539
	1.34	0.6529	3.9729	1.8062	2.7755
	1.35	0.6566	3.9840	1.8198	2.7968
	1.36	0.6615	3.9949	1.8336	2.8180
	1.37	0.6674	4.0055	1.8488	2.8399
	1.38	0.6743	4.0159	1.8645	2.8615
	1.39	0.6819	4.0260	1.8838	2.8838
	1.4	0.6901	4.0360	1.8975	2.9061
Underwater, salinity 13% (actual refractive index n=1.35)	1.33	0.6898	3.9957	1.7931	2.6572
	1.34	0.6903	4.0225	1.7968	2.6445
	1.35	0.6918	4.0487	1.8006	2.6405
	1.36	0.6943	4.0743	1.8060	2.6442
	1.37	0.6977	4.0994	1.8140	2.6865
	1.38	0.7018	4.1239	1.8235	2.6865
	1.39	0.7066	4.1478	1.8353	2.7120
	1.4	0.7121	4.1712	1.8476	2.7386
Underwater, salinity 27% (actual refractive index n=1.37)	1.33	0.8940	4.4589	2.3946	3.4778
	1.34	0.8950	4.4683	2.4003	3.4911
	1.35	0.8967	4.4901	2.4077	3.5040
	1.36	0.8992	4.5114	2.4168	3.5174
	1.37	0.9022	4.5323	2.4266	3.5308
	1.38	0.9058	4.5527	2.4371	3.5445
	1.39	0.9100	4.5727	2.4482	3.5581
	1.4	0.9146	4.5922	2.4604	3.5720
Underwater, salinity 40% (actual refractive index n=1.40)	1.33	0.8038	4.8682	2.1883	3.2080
	1.34	0.8056	4.8881	2.1842	3.2064
	1.35	0.8083	4.9074	2.1812	3.2060
	1.36	0.8116	4.9264	2.1794	3.2086
	1.37	0.8156	4.9449	2.1789	3.2149
	1.38	0.8201	4.9630	2.1799	3.2211
	1.39	0.8251	4.9807	2.1814	3.2275
	1.4	0.8305	4.9980	2.1841	3.2369

**Table 3:** Accuracy evaluation of chessboard cell corner detection using the OpenCV library findChessboardCorners function.

Image Set	Mean	Maximum
In the air	1.7833	3.7317
Underwater (salinity <1%)	2.8408	22.6256
Underwater (salinity 13%)	2.6668	6.7385
Underwater (salinity 27%)	3.3144	11.9810
Underwater (salinity 40%)	4.1901	9.7023

## Data Availability

The data set, as well as the accompanying code for all of the experiments described in the article, are publicly available at https://github.com/Visillect/SaltWaterDistortion (accessed on 14 October 2022).
